# The association between depression and quality of life in the elderly

**DOI:** 10.1093/eurpub/ckac131.125

**Published:** 2022-10-25

**Authors:** A Papageorgiou, M Bakola, KS Kitsou, V Mousafeiris, K Mavridou, P Kallianezos, M Kampouraki, G Charalambous, E Jelastopulu

**Affiliations:** 1 Doctoral Programme in Health Management, School of Health Sciences, Frederick University, Nicosia, Cyprus; 2 Department of Public Health, School of Medicine, University of Patras, Patras, Greece; 3 Department of Orthopedics, Agios Andreas General Hospital of Patras, Patras, Greece; 4 Department of Skin and Venereal Diseases, University Hospital of Ioannina, Ioannina, Greece; 5 Department of Pediatric Surgery, Karamandaneion Children’s Hospital of Patras, Patras, Greece; 6 Nea Madytos Health Center, Thessaloniki, Greece

## Abstract

**Background:**

Depression is the most common cause of emotional disorder in older adults causing functional impairment and leading to lower quality of life (QOL). The aim of the study was to investigate the prevalence of depression, measure the perceived QOL and evaluate the impact of depression on life quality of older people in the community.

**Methods:**

We conducted a cross-sectional study in older people, enrolled in open day care centers and other healthcare facilities. To all participants the Greek validated version of the Geriatric Depression Scale (GDS-15) was applied to screen for depressive symptoms and the EQ-5D-5L scale to estimate their self-reported health status.

**Results:**

A total of 634 seniors participated in the study, 53% were females, mean age 78 years. 45.6% of the participants showed moderate to severe depression. Those who suffered from at least two chronic diseases (75.6%) were more likely to develop moderate or severe depressive symptoms compared to those with only one (19.3%) or none (2.2%) chronic disease. Increased risk of depression was observed in people with Parkinson’s disease (56.5%), mental illness (47.1%), respiratory disease (40%) and stroke (35.1%). Regarding the EQ-5D-5L scale, 72% reported slight to extreme anxiety or depression, 68.2% slight to extreme pain or discomfort, 59.8% slight to extreme problems in performing usual activities, 59.8% in mobility and 40.1% in self-care, respectively. Significant differences were found between people with or without depression. Specifically, participants who reported slight to severe problems in all five domains of the EQ-5D-5L, showed higher levels of depression in the GDS-15.

**Conclusions:**

Our results revealed that elderly with depressive symptoms have a lower quality of life. Given the mutual relationship between chronic diseases, lower life quality and depression, assessment of depressive symptoms are needed in elderly population, mainly in people with multiple chronic diseases.

**Key messages:**

• Depression can be a predictor of poorer quality of life in older population.

